# Successful esophageal bypass surgery in a patient with a large tracheoesophageal fistula following endotracheal stenting and chemoradiotherapy for advanced esophageal cancer: case report

**DOI:** 10.1007/s10388-012-0338-4

**Published:** 2012-07-19

**Authors:** Tatsunori Miyata, Masayuki Watanabe, Yohei Nagai, Masaaki Iwatsuki, Shiro Iwagami, Yoshifumi Baba, Chiyo Furushou, Yoshihiro Ikuta, Tatsuro Yamamoto, Hideo Baba

**Affiliations:** 1 Department of Gastrointestinal Surgery, Graduate School of Medical Sciences, Kumamoto University, 1-1-1, Honjo, Kumamoto, 860-8556 Japan; 2 Department of Anesthesiology, Graduate School of Medical Sciences, Kumamoto University, 1-1-1, Honjo, Kumamoto, 860-8556 Japan

**Keywords:** Tracheoesophageal fistula, Esophageal bypass, Tracheobronchial stent

## Abstract

A 63-year-old man with esophageal achalasia for more than 20 years complained of respiratory distress. He was admitted as an emergency to the referral hospital three months previously. Computed tomography revealed tracheobronchial stenosis due to advanced esophageal cancer with tracheal invasion. He underwent tracheobronchial stenting and chemoradiotherapy. A large tracheoesophageal fistula (TEF) developed after irradiation (18 Gy) and chemotherapy, and he was unable to eat. Thereafter, he was referred to our hospital, where we performed esophageal bypass surgery using a gastric conduit. A percutaneous cardiopulmonary support system was prepared due to the risk of airway obstruction during anesthesia. A small-diameter tracheal tube inserted into the stent achieved ordinary respiratory management. No anesthesia-related problems were encountered. Oral intake commenced on postoperative day 9. He was discharged on postoperative day 23 and was able to take in sustenance orally right up to the last moment of his life. Esophageal bypass under general anesthesia can be performed in patients with large TEF with sufficient preparation for anesthetic management.

## Introduction

Tracheoesophageal fistula (TEF) is a devastating and life-threatening complication of esophageal and bronchogenic carcinomas. It develops in approximately 5–15 % of patients with esophageal malignancies and in less than 1 % of those with bronchogenic carcinoma [1, 2]. Surgical treatment of malignant tumors is usually difficult in this situation, and tracheobronchial or esophageal stenting is frequently indicated to prevent respiratory failure [3]. Esophageal bypass surgery for patients with esophageal cancer is a useful technique to palliate several symptoms, including dysphagia and aspiration pneumonia due to TEF. However, this option may be avoided in cases with large TEFs because of difficulties in respiratory management during general anesthesia. We report a case of esophageal cancer with a large TEF that was successfully treated with surgical esophageal bypass under general anesthesia.

## Case report

**Fig. 1:**
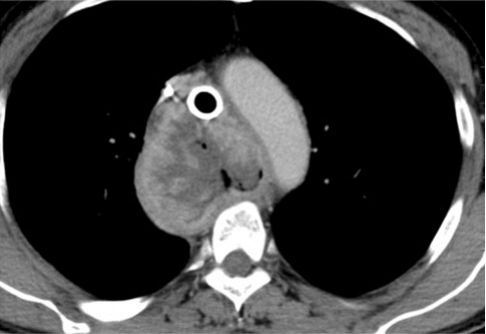
Chest computed tomography (CT) shows a huge esophageal tumor significantly compressing the trachea with the stent

**Fig. 2:**
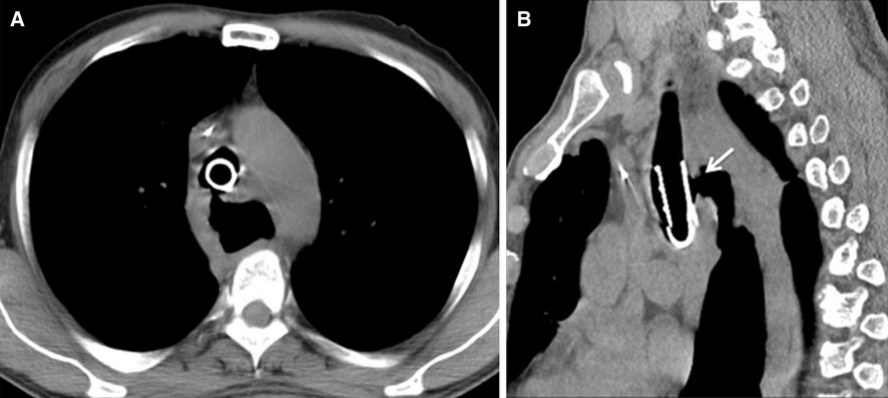
Preoperative chest computed tomography (CT) shows a tracheoesophageal fistula. There is a space around the stent because of chemoradiotherapy (**a**) and a tracheoesophageal fistula at the upper thoracic esophagus (**b**). The *arrow* shows a tracheoesophageal fistula

A 63-year-old man who had suffered from esophageal achalasia for more than 20 years complained of acute respiratory distress due to stenosis of the trachea resulting from advanced esophageal cancer with tracheal invasion. He was admitted as an emergency to the referral hospital and computed tomography revealed a huge esophageal tumor significantly compressing the trachea (Fig. 1). The patient underwent placement of a Dumon Y-shaped tracheobronchial stent (T M Y stent:φcon) to avoid suffocation. He was diagnosed as having esophageal cancer with tracheal invasion, and then underwent chemoradiation therapy (CRT). Following 18 Gy of irradiation and a course of chemotherapy using docetaxel, cisplatin, and 5-fluorouracil, a large TEF developed despite significant tumor shrinkage (Fig. 2a, b). The physician decided to cease the CRT and treat him with the best supportive care. Oral intake was prevented due to aspiration through the TEF, and a gastrostomy was performed under endoscopy.

The patient was referred to our hospital as he was unable to eat. At the time of admission, he had a good performance status and no comorbidities. We initially considered the possibility of esophageal stenting to close the TEF. However, as CRT significantly decreased the size of the esophageal tumor, and there had been an esophageal dilatation due to pre-existing achalasia, an esophageal stent seemed unlikely to be able to close the TEF. Therefore, we decided to perform an esophageal bypass using a gastric conduit after obtaining informed consent.

We prepared a percutaneous cardiopulmonary support (PCPS) system that could be used whenever needed, and both arterial and venous catheter sheaths were inserted from the femoral vessels prior to an endotracheal intubation. We then intubated a 6.0 mm spiral tube under the guidance of both bronchoscopy and X-ray guidance. A cuff of the tracheal tube effectively sealed the space between the stent and tracheal tube and enabled positive pressure ventilation. We were then able to perform esophageal bypass without using PCPS. Esophageal bypass surgery was performed through upper abdominal and collar neck incisions. Bypass was made using a gastric conduit through the subcutaneous route, and esophagogastrostomy was performed using a circular stapler. A decompression tube was inserted into the esophagus through the distal stump covered with the greater omentum. After the surgery was completed, the tracheal tube was safely removed under bronchoscopy. The patient was able to start oral intake on postoperative day 9, and was discharged on postoperative day 23. Though he died of respiratory failure three months after the operation, he was able to take in sustenance orally and to live at home after the operation.

## Discussion

The prognosis for patients with a malignant TEF due to esophageal cancer is reported to be extremely poor [4]. In patients with TEF, oral intake is limited by paroxysmal coughing, which results in malnutrition. In addition, recurrent pulmonary infections and sepsis may become direct causes of death. Therefore, closure of the TEF is much more important than treating the underlying malignancy. However, in this case, the TEF was too large to close and the patient was treated with the best supportive care. The most effective treatments for malignant TEF are esophageal stenting and esophageal bypass surgery. The covered expandable metallic stent is emerging as a superior alternative to unexpandable esophageal prostheses. A Dumon Y-shaped tracheobronchial stent had already been inserted. Although this stent succeeded in keeping the airway open, it was ineffective at closing the TEF. Esophageal stenting is normally considered to be one of the treatment choices, but it seemed to be ineffective in this case because of the enlarged esophageal caliber due to pre-existing achalasia. Indication for bypass surgery differs among patients. In this case, curative resection of the esophageal tumor was impossible. There was no other way to enable oral intake than esophageal bypass surgery, although the patient strongly wished to eat. In addition, his general condition was sufficient for surgery and his estimated life expectancy was more than three months.

There are several reports concerning the usefulness of esophageal bypass for malignant TEFs [5, 6]. However, cases with a large TEF must be rare, because there is no description of anesthetic management in these articles. Meunier et al. [5] reported that in their series, one patient with a large TEF died just after surgery due to airway obstruction. The main issue in our particular case was whether we could perform general anesthesia safely. Firstly, an endotracheal intubation might cause migration of the stent, resulting in suffocation. Second, positive pressure ventilation might become insufficient during the operation. Third, migration of the stent might also occur when the tracheal tube is removed. We discussed all of these problems with anesthetists and cardiovascular surgeons, and concluded that we should prepare the PCPS so that it could be used whenever needed. Fortunately, ordinary respiratory management was achieved by several devices. We used a small-diameter tracheal tube which was inserted into the tracheobronchial stent. The cuff of the tube successfully sealed the space between the tracheal tube and the stent. In order to avoid stent migration, we used bronchoscopy and X-ray imaging for guidance during both intubation and extubation.

In conclusion, an esophageal bypass can be performed under general anesthesia even in patients with a large TEF, although sufficient preparation for anesthetic management, including the preparation of the PCPS, is needed.
